# Knowledge translation and integration of research evidence into practice: A national mandate

**DOI:** 10.4102/safp.v67i1.6158

**Published:** 2025-11-12

**Authors:** John Musonda, Deidre Pretorius, Christiaan Visser, Olufemi Omole

**Affiliations:** 1 Department of Family Medicine and Primary Care, School of Clinical Medicine, Faculty of Health Sciences, University of the Witwatersrand, Johannesburg, South Africa

**Keywords:** knowledge translation, research evidence translation, evidence-based tools, research findings, research alignment, research implementation, family medicine

## Abstract

New medical knowledge is being rapidly added to the existing body of scientific understanding. However, the degree to which knowledge translation and the integration of research evidence into clinical practice are aligned and implemented remains unclear. This paper aims to promote the implementation of research evidence, thereby influencing professional behaviors, patient outcomes, and health policy. The focus is on the district research outcomes to facilitate changes in practice. An analysis of the dissemination of research findings within the health district is presented. A locally designed, innovative model is introduced, and global evidence-based models are incorporated to provide further insights. We conclude by reaffirming that utilizing evidence-based tools to develop and implement clinical guidelines and policies could transform practice and enhance patient outcomes.

**Contribution:**

The article’s contribution is in motivating health care workers to engage in research to expand their competencies, and promote the alignment of research findings with implementation.

## Introduction

Medical research is considered crucial by many professionals for expanding knowledge, advancing the understanding of various topics, optimising resource utilisation, enhancing technologies and processes, and improving patient outcomes.^1^ Hence, research may provide solutions to real-world problems. Nonetheless, does our research translate into improved quality of care and/or better patient outcomes? In addition, it raises the question of whether research addresses the country’s health priorities.

Globally, health research seldom translates into clinical practice; if it does, it is driven more by individual factors than by organisational factors.^1^ The problem is widely diverse and increasing in Africa because of reported disparities in research findings, dissemination, knowledge translation (KT), and the integration into clinical practice.^2^ There seems to be no precise alignment between the application of research findings and clinical practice, generally.^3^

Khumalo et al. conducted a qualitative review in KwaZulu-Natal, South Africa, and reported that 72% of the thematic areas identified in the findings did not align with health research priorities.^4^ The dissemination of scientific knowledge to the intended audience is imperative; therefore, communicating validated research information constitutes an essential element of KT. The clinical research conducted must address the health priorities previously identified in the health care system.^2^ Thus, when practitioners, academics, the public, and stakeholders collaborate to promote deeper engagement, there is evidence that the research-clinical practice gap can be overcome.^2,5,6^

## Knowledge translation and integration into family practice in a local health district

**FIGURE 1:**
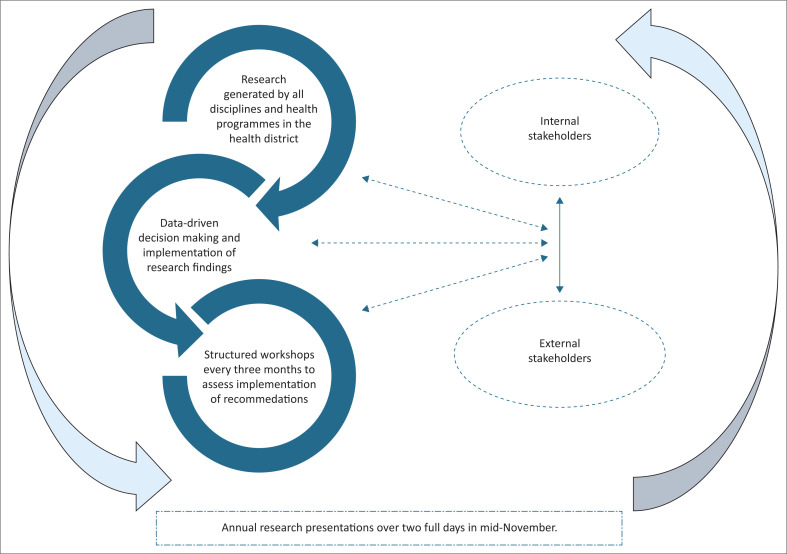
An innovation to disseminate and integrate research evidence into practice in a health district in Gauteng, South Africa.

The health district developed a locally relevant model (Figure 1) of KT and integration into family practice by learning from contextual experiences and evidence.

To demonstrate the implementation of the model (Figure 1), a cervical cancer screening study from the Ekurhuleni health district is used. In South Africa, cervical cancer remains the second-highest cancer in women.^7^ The cervical cancer screening coverage in Ekurhuleni health district declined from 51% in 2018 to 45% in 2020, and the prevalence of human papillomavirus (HPV) infection in women living with HIV and AIDS (WLWHA) remains high.^8^

A retrospective, descriptive, cross-sectional study evaluated cervical cancer screening among women living with HIV and attending primary care clinics.^9^ A cluster randomisation and systematic selection of 93 clinics in a densely populated area yielded 550 completed clinical records for review. The calculated sample size was 596, and the analysis was conducted using Stata version 16.1.^9^ The study concluded that there was poor uptake of cervical cancer screening and delays in initiating screening. The recommendations focused on accelerated primary health care (PHC) staff training led by public sector family physicians.^9^

The key enablers were effective communication and collaboration between the researchers and internal and external stakeholders.^1,3,6^ The researchers’ immediate target audience at the annual district research conference was attendees, including medical practitioners, academics, PHC nurse clinicians, health managers, supporting partners, and other decision-makers. The district had hosted an annual research conference for over two decades, and family physicians actively participated in organising them. The environment created a dynamic engagement among stakeholders.

Dissemination of research findings to clinics, health promotion, and structured meetings and workshops with staff were other methods used for KT. The social media platform and health promotion to the public, as well as staff presentations, were crucial communication channels. When conducted appropriately, disseminating research findings enables transparency and builds trust between researchers and users of the evidence through training and workshops.^5,6^ It was a non-linear process involving data-driven decision-making and intervention implementation, which differed from the usual top-to-bottom approach. The approach improved cervical cancer screening coverage in the district, but the coronavirus disease 2019 (COVID-19) pandemic hampered it. However, the KT and integration activities remain sustained.

Disseminating research findings can be a complex process, as several challenges must be addressed. Therefore, to translate and integrate research evidence into practice, communicating the meaning and concepts of how people exchange information is key to collaboration and cooperation among internal and external stakeholders.^1,5^ Our model is not a series of simple steps but a structured, dynamic, and interconnected process. It is dynamic because it is flexible, adaptable, and evolves in response to new information. However, the interconnectedness refers to the relationships that depend on one another to create a holistic nature. Hence, stakeholders are assisted in understanding how research knowledge is integrated into practice and influences personal, professional, and public behaviours to improve patient outcomes and clinical impact.^1,5,6^

## Global evidence-based strategies for knowledge translation and integrating research evidence into family practice

Evidence shows that the KT and integration process applies research findings to transform them into actionable activities aimed at changing clinicians’ behaviour and practice. However, compelling strategies and messages must be designed and disseminated widely using evidence-based tools and structured frameworks or models.^6,10^

### Knowledge-to-Action (KTA) Framework

This systematic approach helps researchers apply research knowledge in practical ways. The KTA Framework is not linear but instead consists of a sequence of activities, support, and interconnected steps that transfer research knowledge into clinical practice at any level of care. It is broadly divided into two components: the knowledge creation steps (Phase 1) and the application or action (Phase 2).^6,10^

After the research study is completed and disseminated, knowledge creation (phase one) begins by identifying areas that need improvement. That is based on challenges in operational structures, processes, and outcomes through a knowledge inquiry. The next step in the KTA framework is knowledge synthesis, which combines components or parts of the framework to form a whole connected meaning and specify what needs to be changed.

The Action or Application (phase two) identifies and reviews problems using the best available evidence in the literature. Knowledge of what needs to change in the clinical practice should be explicit. A hierarchy must be followed to ensure the essential steps are completed before proceeding to the next step, which can prevent delays and failures in implementation.^6,10^ An implementation plan should include objectives and specific, measurable outcomes, such as activities, performance targets, and timelines. The multidisciplinary clinical team should drive the desired change in clinical practice. The above interventions could ensure that resources, tools, and protocols are utilised effectively to transfer knowledge from research evidence to practice. Essentially, clinical experience, scientific knowledge, and patient preferences are crucial for practising evidence-based medicine.^10,11^

### The Revised Iowa Model

The model is summarised as an evidence-based algorithm (Figure 1) that is easy to follow.^12,13^ It begins by identifying opportunities and priorities, helping team formation, and gathering research evidence. The focus is on bringing together all research evidence or validated information components to support a targeted improvement project in clinical practice. The implementation team should design and pilot the intended clinical practice behaviours. If the change is deemed appropriate for adoption, an implementation plan must be developed like the KTA framework. The interventions are monitored by measuring performance targets and outcomes from improvement projects.^13,14^

**FIGURE 2:**
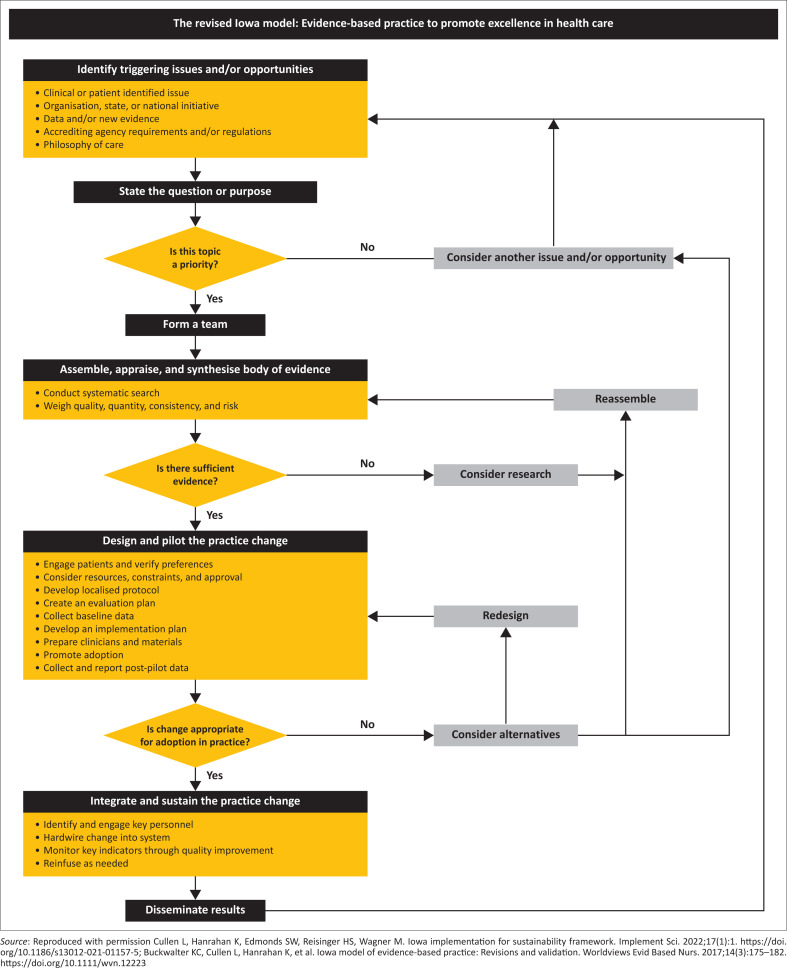
Revised Iowa Model to integrate research evidence into clinical practice to promote excellence in health care.

The Revised Iowa Model^13^ can enhance and change clinicians’ behaviour and influence health policy development and implementation. It is relevant, user-friendly, and has implications for improved primary health care and family practice at the endpoint.^15^ The model is especially valuable for its focus on frontline practice issues and its requirement that proposed changes should align with organisational priorities. Through its focused approach, the model increases the likelihood of successful and sustained implementation of evidence-based clinical practices (See Figure 2).

### Collaboration with internal and external stakeholders

To achieve a successful KT, it is essential to collaborate with stakeholders who utilise the knowledge, such as practitioners, policymakers, health managers, the public, and other relevant key players. The first step would be to identify the stakeholders and then guide them to ensure they are trained and supported in achieving the goals and vision of improving health services through evidence-based practice.^14^ Organisational and individual managers and leaders should be encouraged to facilitate and direct the process and, in turn, capacitate others to lead similar processes.^16^

### Contextualising KT into clinical practice

A fit-for-purpose approach to the local environment, including its culture and aspirations, must exist. National, provincial, and district research priority-setting must ensure that research aligns with its purpose and is relevant to the community’s culture and needs.^4^ In the national health research strategy document, which guides research priorities in South Africa, a structured process exists to bridge the gap between research and clinical practice.^17^ That is important because scientific research must be aligned with people’s needs.

### Monitoring and evaluating the intended changes

Tracking targets and aligning research evidence and practical activities are essential in monitoring performance^6^ and maintaining the balance between translating knowledge and research evidence into family practice. Family practice must respond to people’s needs and develop and strengthen family medicine through research activities.

## Reflections on using models for knowledge translation and integration into practice

Both evidence-based tools enable the effective translation of research knowledge into practice. The KTA Framework is a comprehensive process that will help identify research gaps, disseminate information, and implement practical changes in PHC and family medicine.^10,11^ The Iowa model, first of all, identifies gaps, followed by the research process. The health care system in Gauteng serves under-resourced communities, and research is not always directly linked to known challenges.^18,19^ Research is often driven by personal interest or post-graduate studies. This makes the locally developed model a relevant model, as known challenges and hidden issues can be identified and addressed.

As family physicians practise, research forms a strong component of their leadership and governance role, including clinical supervision, audits, and quality improvements.^15,16^

Internal and external challenges exist at the individual, health system, and organisational levels. Those ought to be reasonably managed by experienced team members, and could include inadequate knowledge, incompetence, and time and resource constraints.^1^

## Conclusion

The dissemination of research findings, KT, and integration into clinical practice among internal and external stakeholders, as well as other relevant parties, could promote synergy, alignment, and bridge the disparity between research evidence and practice. Among the competencies of family physicians are research, KT, and the implementation of research evidence into practice. Family physicians are encouraged to bridge the research-clinical practice gap, as it is essential to ensure that research findings are disseminated, translated, and effectively integrated into clinical practice, thereby promoting understanding, trust, and their application. The article contributes to a broader discourse on KT because it is practical, evidence-based, and viewed from the perspective of family practice. Further research is recommended to identify barriers to knowledge translation in low-resource settings as well as to evaluate specific research strategies, such as telehealth and peer networks. In addition, we recommend conducting research on the feasibility of competency-based KT training programmes for primary care teams.
